# Successful regenerative endodontic treatment in a tooth with incomplete root apex and posttreatment apical periodontitis: A case report 

**DOI:** 10.4317/jced.59358

**Published:** 2022-06-01

**Authors:** Renato Lenzi, Karen Brisson-Suárez, Alessandra Baasch, Flávio R. F. Alves, Isabela N. Rôças, José F. Siqueira Jr 1,3,5

**Affiliations:** 1 Member of the EndoChat research group, Rio de Janeiro, Brazil 2 Private practice, Rio de Janeiro, Brazil; 2 Private practice, Rio de Janeiro, Brazil; 3 Postgraduate Program in Dentistry, University of Grande Rio (UNIGRANRIO), Rio de Janeiro, Brazil; 4 Department of Endodontics, Santa María University, Caracas, Venezuela; 5 Department of Dental Research, Faculty of Dentistry, Iguaçu University (UNIG), Nova Iguaçu, Brazil

## Abstract

This case report describes the procedure and outcome of regenerative endodontic treatment (RET) in a tooth with incomplete root apex and posttreatment apical periodontitis. A 44-year-old patient was referred to the endodontist because of a periapical lesion on tooth #21 and a recent episode of acute periapical abscess. On clinical and radiographic examination, this tooth presented with tenderness to percussion and palpation, periapical radiolucent lesion, external apical resorption, and incomplete apex formation. After coronal access, the filling material was removed, and the canal was gently prepared with hand files, using 1% NaOCl as the main irrigant followed by final irrigation with 17% EDTA, activated with XP-endo Finisher (FKG Dentaire, La Chaux-de-Fonds, Switzerland). The root canal was filled with a double antibiotic paste with ciprofloxacin and metronidazole (1:1). After three weeks, RET was performed by stimulating bleeding into the canal, and when a clot was formed, a bioceramic (EndoSequence BC Sealer, Brasseler USA, Savannah, GA) plug was placed on it, followed by coronal restoration. The tooth remained asymptomatic since RET was concluded. Clinical and radiographic follow-ups showed complete repair of the apical periodontitis lesion and the absence of symptoms after eight months. This satisfactory outcome was confirmed after 34 months.

** Key words:**Bioceramic material; ciprofloxacin; metronidazole; persistent apical periodontitis; regenerative endodontic treatment.

## Introduction

Pulp necrosis and infection in immature teeth occur primarily because of caries, trauma, or dental anomalies such as dens invaginatus (1). Different approaches have been indicated to stimulate or create conditions to permit further root development or promote apical closure. The root walls of immature teeth can be too thin, making them fragile and susceptible to fracture. This condition is of especial concern because the enlargement produced by canal preparation and loads generated during filling procedures could weaken and make the tooth prone to root fracture (2).

Regenerative endodontic treatment (RET) has demonstrated promising outcomes, with potential to stimulate continued root formation (length and thickness), reestablish tooth sensitive function, and promote healing of apical periodontitis (3). This treatment modality is based on three approaches: (i) root canal disinfection, (ii) scaffold provision, and (iii) coronal sealing (4).

RET has become the first treatment choice for immature teeth with pulp necrosis (5) and has also been considered an option for mature teeth with necrotic pulps and sizeable apical diameter (6). Furthermore, recent reports showed the potential of this procedure to be used to retreat teeth with posttreatment apical periodontitis (7). Although tooth sensitivity could not be reestablished, apical periodontitis and symptoms were resolved in these reports.

The present case report describes the procedures and outcome of RET in a tooth with persistent apical periodontitis after previous root canal treatment.

## Case Report

A 44-year-old female patient with noncontributory medical history was referred to an endodontist (R. L.). According to the report, she had developed an acute alveolar abscess in the left maxillary anterior area one month before. The abscess had been treated with systemic antibiotics (amoxicillin, 875 mg every 12 hours, for 7 days) and analgesics (sodic dipyrone, 1 g). Dental history revealed a previous traumatic injury in the anterior maxillary teeth at the age of 12 years. Subsequently to trauma, conventional root canal therapy was performed in tooth #21, and at the age of 34 years, it was retreated.

**Figure 1:**
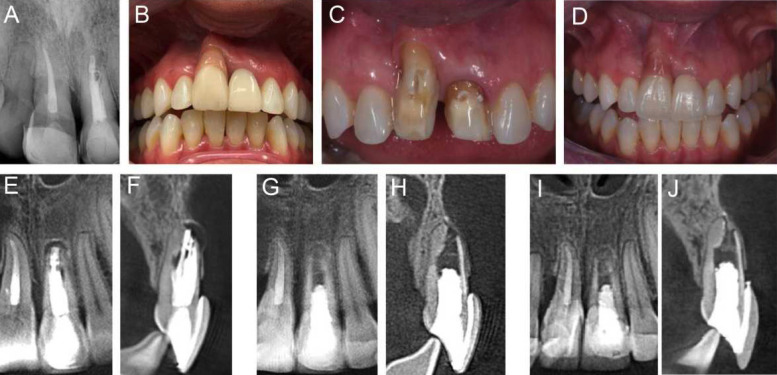
A) Initial periapical radiograph showing a sizeable inadequately-filled canal space associated with a discrete periapical radiolucent lesion on tooth # 21. B) Preoperative clinical aspect. C,D) Coronal reconstruction of teeth #8 and #9 with esthetic restoration. E, F) Preoperative cone-beam computed tomography (CBCT) images. Teeth #9 showed conventional root canal therapy and showed a large inadequately filled canal space associated with a periapical radiolucent lesion. Postoperative CBCT of tooth #9 eight months (G,H) and 34 months (I,J) after RET showing complete repair of the apical periodontitis lesion.

On clinical examination, tooth #21 presented full crown coverage and tenderness to percussion and palpation. Periapical radiographs showed a large poorly-filled canal space associated with a discrete periapical radiolucent lesion confirmed by cone-beam computed tomography (CBCT), which also revealed a slightly overextended root canal filling, external apical root resorption, and incomplete apex formation of tooth #21 (Fig. 1). Tooth #11 had also been previously endodontically treated, presenting with a slightly darkened crown, but no apical periodontitis.

RET was conducted in tooth #21 initiated by local anesthesia with 2% lidocaine containing 1:100,000 epinephrine and rubber dam isolation. After conventional coronal access, the root canal filling material was removed using hand Hedstrom files under copious irrigation with 5.25% NaOCl. The working length was established at 0.5 mm short of the radiographic root apex. The canal was prepared using hand files with short and gentle movements to avoid over-enlargement of the already thin dentinal walls. A final rinse was performed with 10 mL 17% EDTA, which was agitated with the XP-endo Finisher instrument (FKG Dentaire, La Chaux-de-Fonds, Switzerland) (8,9). The root canal was dried with sterile paper points, and a double antibiotic paste with ciprofloxacin and metronidazole (1:1) was used as an intracanal medication. Finally, the access cavity was temporarily sealed.

After 3 weeks, the patient returned and the tooth was anesthetized with 2% lidocaine without vasoconstrictor, isolated with a rubber dam, and accessed. The intracanal antibiotic paste was removed using a hand K-file and irrigation with 20 mL 1% NaOCl. A final rinse was performed with 10 mL 17% EDTA. Next, bleeding was induced by passing a size 30 K-file 2 mm beyond the apex. After an intracanal clot was formed, a bioceramic (EndoSequence BC Sealer, Brasseler USA, Savannah, GA, USA) plug was placed in the coronal third of the root canal, followed by a permanent coronal restoration using composite resin.

The tooth remained asymptomatic since RET was concluded. Clinical and radiographic follow-ups showed the complete repair of the apical periodontitis lesion and the absence of signs or symptoms of inflammation after 8 months (Fig. 1). After 34 months, the tooth was still asymptomatic, and the periapical tissues were radiographically normal, with newly formed bone visible at the very apical part of the apical foramen (Fig. 1).

## Discussion

Persistent intraradicular or extraradicular infections are the major causes of posttreatment apical periodontitis (10). Therefore, infection control plays a pivotal role in conventional and regenerative endodontic therapies. Besides infection control, RET protocols should induce the release of growth factors from the dentin wall to stimulate ingrowing cells to produce new dentin and/or cementum to increase the root length and thickness, or induce apical closure.

RET is mostly indicated for immature teeth with necrotic pulps and apical periodontitis, with more predictable results in young patients (11). Dental trauma injury such as avulsion and luxation may damage the apical papilla and the Hertwig epithelial root sheath, and predispose to a lower success rate of RET (12). Successful regeneration procedures may depend on the size of the apical foramen,which permits an adequate influx of blood and regenerative/reparative cells in the root canal. Apical diameters varying from 0.5 to 1.0 mm resulted in the highest success rates after RETs (13). In the present case, the size of the apical opening was very large because root formation was incomplete.

In this case report, irrigation was performed with 1% NaOCl since it has reduced cytotoxic effects and less denaturing effects on the dentinal walls when compared to higher concentrations, mainly when EDTA is employed as the final rinse (14). The final rinse was performed with 17% EDTA to remove the smear layer and induce the release of growth factors from dentin (15). Although a triple antibiotic paste has been commonly used in RET procedures, the clinician opted for not including minocycline, because of the risk of causing tooth staining (16). The XP-endo Finisher instrument was included in the treatment protocol to drive the irrigant solutions to areas of difficult access in the canal system (8,9).

Although the American Association of Endodontists recommends the use of low concentration antibiotic pastes or calcium hydroxide as options for RET, the European Society of Endodontology recommends calcium hydroxide as the first option because of some drawbacks related to the antibiotic pastes, including tooth discoloration, cytotoxicity, sensitization, development of resistance and difficulty of removal from the root canal. However, further studies are necessary to confirm the real impact of these issues, considering that the medication is confined to the root canal.

Evidence suggests that proper regeneration of the pulp-dentin complex does not occur after RETs, and histologic analyses revealed cementum-like tissue and uninflamed fibrous connective tissue inside the root canal (17). Although the pulp replacement tissues are not true pulp tissue, they are vital tissues with innate and adaptative immune defense mechanisms innervated by sensory fibers (18). However, it is very difficult to ascertain the conditions of the tissue in the root canal in the clinical setting.

## Conclusions

This case report shows that RET was successfully used to retreat a tooth with incomplete apex formation and posttreatment apical periodontitis. This approach led to a favorable and sTable outcome for almost 3 years, represented by the elimination of signs and symptoms and periradicular tissue healing. Continued root formation and thickening of the canal walls are generally not expected in cases like this. Randomized, prospective trials, with long-term follow-up examination, are required to establish if RET is a viable alternative to conventional root canal treatment/treatment to deal with infected mature teeth associated with apical periodontitis.
